# The Dublin Meeting: "Facts Turned into Virtual Falsehoods"?

**Published:** 1919-03-15

**Authors:** Vera Matheson

**Affiliations:** 20 Fitzwilliam Square, Dublin


### THE EDITOR'S LETTER-BOX.

**[Correspondence on all subjects is invited, but we cannot in any way be responsible for the opinions expressed by our correspondents, who must give their name and address as a guarantee of good faith, but not necessarily for publication. Correspondents are reminded that brevity of style and conciseness of statement greatly facilitate early insertion.]**

### THE DUBLIN MEETING: "FACTS TURNED INTO VIRTUAL FALSEHOODS"?

*To the Editor of* The Hospital.

Sir,—It is always a pity when facts are misrepresented, and it is especially so in regard to Ireland, for the English people as a whole know so little about us that they naturally have to believe what they are told. I do not know if "Ierne" was present at the meeting in the Dublin Mansion House on February 28, on which he comments in your current issue, but I was present, and it seems only fair to the nurses of Dublin to state exactly what happened. I went to the Mansion House fully expecting great enthusiasm in favour of a trades union for nurses. The meeting was crowded with nurses—it was wonderful that so many should have been able to come at the same hour at a time when the influenza epidemic is raging furiously and people are crying out in vain for a trained nurse. The speeches and arguments by trades-union delegates were excellent, there was no fault to find with them; and the speech of the delegate from the Teachers' Union was particularly telling. The only point to raise was that the speakers completely ignored the progress that has been taking place in nursing matters in the last two or three years, quietly and without any flourish of trumpets through professional channels, and chose to attribute the course of the current of public opinion to the talk of a trade union. Yet in spite of the crowded room, in spite of the telling speeches, in spite of the excellent arguments, when the trades-union delegates had finished speaking and the meeting was thrown open for discussion, not a single nurse got up to welcome the long-delayed millennium. Several nurses spoke against it, and even keen prejudice against the College of Nursing did not prevent the audience from loudly applauding the college member who objected to handing over her personal responsibility towards her patients to a committee of nurses or any one else. All this might be attributed to other causes, but when Miss Bennett called for a show of hands in favour of a trade union for nurses, to my amazement, instead of the unanimous uplifting of arms which I had expected, there were not a dozen in the body of the hall, and one had to look carefully round the walls to find them. There was no doubt about its being a small minority of the audience, and Miss Bennett, who was undeniably disinterested and only anxious to help, looked decidedly disappointed.

I make no comment upon this result, nor upon "Ierne's" sublime contempt for Irish efforts towards reform, but it certainly seems a pity to apply so much imagination to facts as to turn them into virtual falsehoods, and I therefore beg a little of your valuable space for this plain statement of what took place.

Yours, etc.,

Vera Matheson.

20 Fitzwilliam Square, Dublin,

March 8, 1919.

